# Initial assessment of femoral proximal fracture and acute hip arthritis using pocket-sized ultrasound: a prospective observational study in a primary care setting in Japan

**DOI:** 10.1186/s12891-020-03326-x

**Published:** 2020-05-11

**Authors:** Takashi Akimoto, Tadashi Kobayashi, Hiroki Maita, Hiroshi Osawa, Hiroyuki Kato

**Affiliations:** 1 Department of General Medicine, Hirosaki University School of Medicine and Hospital, 53 hon-cho, Hirosaki-shi, Aomori, Japan; 2 General Medicine, Hirosaki University Graduate School of Medicine, 53 hon-cho, Hirosaki-shi, Aomori, Japan; 3 Development of Community Healthcare, Hirosaki University Graduate School of Medicine, 53 hon-cho, Hirosaki-shi, Aomori, Japan

**Keywords:** Acute hip pain, Diagnostic accuracy, Elderly care, Hip arthritis, Hip fracture, Point-of-care ultrasound

## Abstract

**Background:**

Acute hip pain caused by femoral proximal fractures or acute hip arthritis requires imaging for accurate diagnosis. Although pocket-sized ultrasound (PsUS) offers several advantages over other imaging modalities, there is limited information regarding its use in diagnosing femoral proximal fractures or acute hip arthritis. Thus, we aimed to validate the diagnostic accuracy of PsUS for both disorders.

**Methods:**

In this prospective observational study, outpatients with acute hip pain were diagnosed according to a fixed procedure of the PsUS probe handling. We verified the diagnostic accuracy of PsUS findings (cortical discontinuity and joint fluid retention) and compared it with that of radiography, computed tomography, and magnetic resonance imaging.

**Results:**

Our study included 52 outpatients (mean age, 78.0 years; female, 88.5%). Of 26 patients diagnosed with femoral proximal fractures, 14 had femoral neck fractures and 12 had femoral trochanteric fractures. The sensitivity and specificity for identifying cortical discontinuity in femoral proximal fractures were 0.96 and 0.92, respectively. The sensitivity for identifying either cortical discontinuity or joint fluid retention in femoral proximal fractures or acute hip arthritis was 0.97.

**Conclusions:**

Negative PsUS findings of cortical discontinuity and joint fluid retention in the hip are useful for ruling out femoral proximal fractures and acute hip arthritis. PsUS and radiography have comparable diagnostic accuracies, and PsUS could aid in the initial assessment of acute hip pain among the elderly in primary care settings.

## Background

Acute hip pain hampers the activities of daily living in the elderly and is classified, based on its location, as anterior, posterior, and lateral hip pain [1]. There are various causes of acute anterior hip pain, including femoral proximal fractures and acute hip arthritis. Femoral proximal fracture (e.g., a femoral neck or femoral trochanteric region fracture) commonly causes acute hip pain, resulting in healthy elderly individuals often becoming bedridden. Acute hip arthritis (e.g., acute aseptic arthritis or acute septic arthritis) [2] is a clinical syndrome characterized by acute onset of hip pain and joint fluid retention. Therefore, in addition to orthopedic physicians, primary care physicians need to perform appropriate initial assessments of hip pathologies.

Femoral proximal fracture often requires surgery and is generally diagnosed using radiography and, occasionally, computed tomography (CT), which reveal an intra-articular hematoma with cortical discontinuity. Acute hip arthritis, except infectious arthritis, does not require surgery and is often revealed on radiography and magnetic resonance imaging (MRI) as joint fluid retention. However, physicians cannot always use these large and expensive devices in rural clinics or nursing homes. The diagnostic accuracy of ultrasound devices (US) is comparable to that of radiography devices [3, 4]. Recently, US devices have become more affordable, smaller, and more portable. They have often been called next-generation stethoscopes [5, 6]. Physicians use hand-carried US devices to diagnose fractures in emergency rooms and outpatient departments [7, 8]. Only one case report was reported using US for femoral proximal fracture [9].

Pocket-sized US (PsUS) devices have also been used in various settings, including clinics and nursing homes. Currently, US is expected to become an imaging modality to diagnose fractures and arthritis in all medical-care settings.

Most physicians use PsUS devices for abdominal, obstetric, cardiac, and musculoskeletal examinations [10, 11]. The current study shows that PsUS is used by physicians in the primary care setting. However, to our knowledge, there has been only one report of radial fractures diagnosed using PsUS [12]. Further, there are no reports of diagnosis of acute hip arthritis using PsUS. Therefore, to evaluate the diagnostic contribution of PsUS during the initial assessment of acute hip pain in the elderly, we assessed the sensitivity (Sn) and specificity (Sp) of PsUS findings (cortical discontinuity and joint fluid retention) in diagnosing femoral proximal fractures (femoral head, femoral neck, and femoral trochanteric region fractures) [13] and acute hip arthritis.

## Methods

This study was approved by the medical ethics committees of Hirosaki University (2015–260, 2nd April, 2015) and National Hospital Organization Morioka Hospital (Morioka Hospital) (2015–10, 1st February, 2015). This study included patients with acute hip pain who visited the emergency outpatient unit at Morioka Hospital, which provides primary care in the area, from April 2016 to March 2017. We obtained informed consent from all patients. We collected the following information in the clinical examination room: 1) basic characteristics (age, sex, and oral medication, including antiplatelet and anticoagulant medications); 2) PsUS findings of cortical discontinuity (a direct finding of fracture) and joint fluid retention (a direct finding of hematoma or fluid 6 mm above the distance between the anterior aspect of the femoral neck and the internal aspect of the anterior joint capsule at the femoral neck long axis [14]); and 3) radiographic, CT, and MRI findings following PsUS examination.

**Fig. 1:**
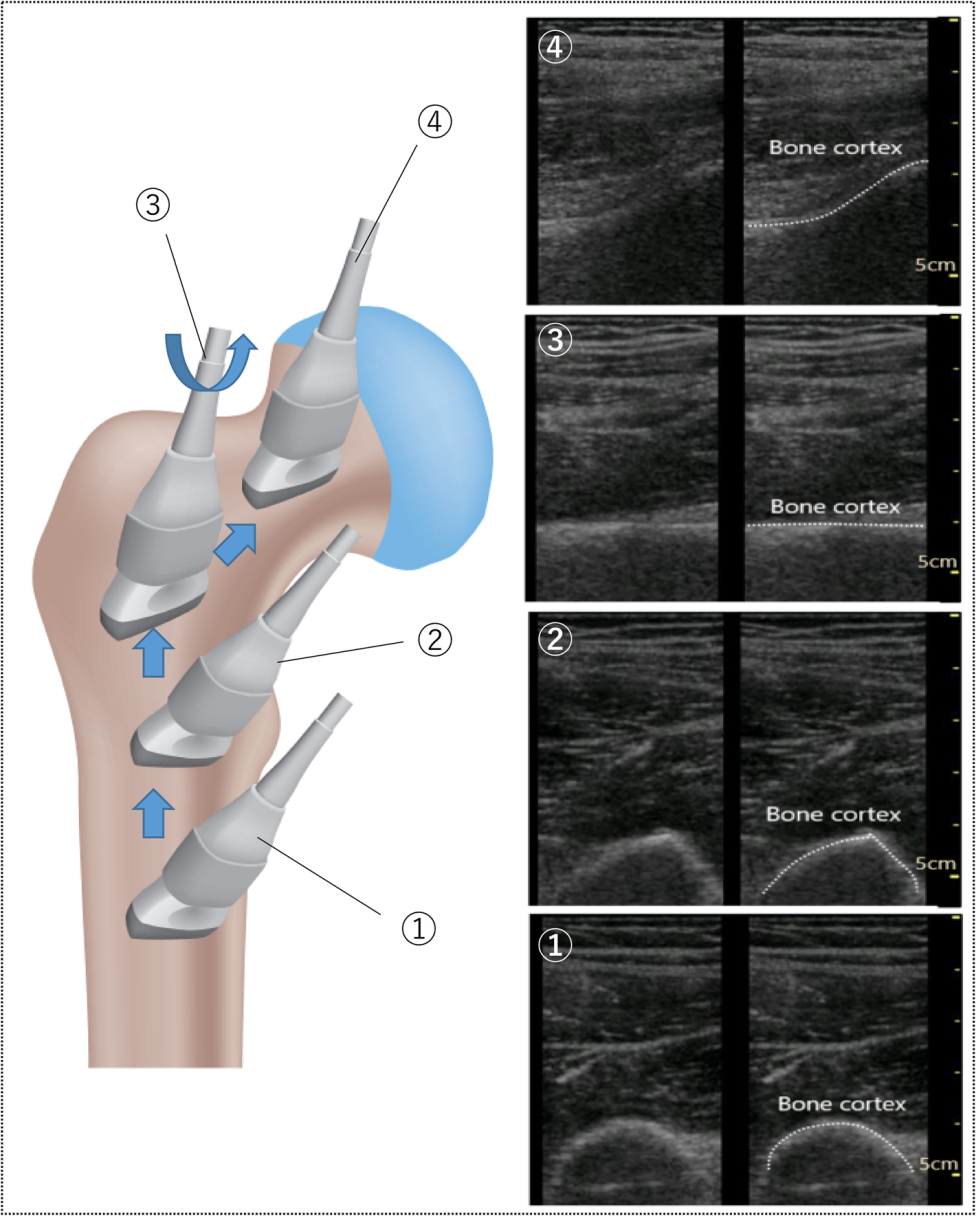
Our stylized handling of the ultrasound probe to detect hip joint fracture. The ultrasound probe was moved in the order from ① to ④. This figure was drawn up by TK (author)

We used the linear-type probe of the Vscan dual probe (GE Vingmed Ultrasound AS, Horten, Norway), one of the most widely used PsUS devices in Japan. An examiner experienced in the use of PsUS (TA; the first author; 11 years of experience) performed the examinations with our stylized handling method to detect cortical discontinuity and joint fluid retention (Fig. 1), as follows: (1) the probe was set in the proximal position of the affected thigh to view the short axis image of the femoral bone trunk; (2) the probe was slid in the proximal direction of the femur while maintaining the short axis view; (3) the probe was rotated at the position at which the shape of the femur changes at the lesser trochanter of the femur, and the femoral neck long axis image of the femur was viewed, checking for extra-articular fractures (femoral trochanteric region fracture); (4) the probe was slid in the direction of the femoral head to view the long axis image of the femoral neck, checking for intra-articular fractures (femoral head fracture, femoral neck fracture) and acute hip arthritis.

We evaluated the diagnostic accuracy (Sn and Sp) of cortical discontinuity and joint fluid retention assessed using PsUS by comparing it with radiography, CT, and MRI. All diagnoses from radiography, CT, and MRI were independently confirmed by a blinded radiologist. We analyzed our data using Fisher’s exact test and 2-way contingency table analysis. Statistical significance was defined as *P* < 0.05.

The sample size of our study was calculated from our clinical experience (α error, 0.05; power, 0.9; Sn, 0.8; Sp, 0.7). The required sample size was 46. We used EZR version 1.30 (Saitama Medical Center, Jichi Medical University, Saitama, Japan) statistical software for all analyses, which is a modified version of R Commander that includes statistical functions frequently used in biostatistics [15].

## Results

**Fig. 2:**
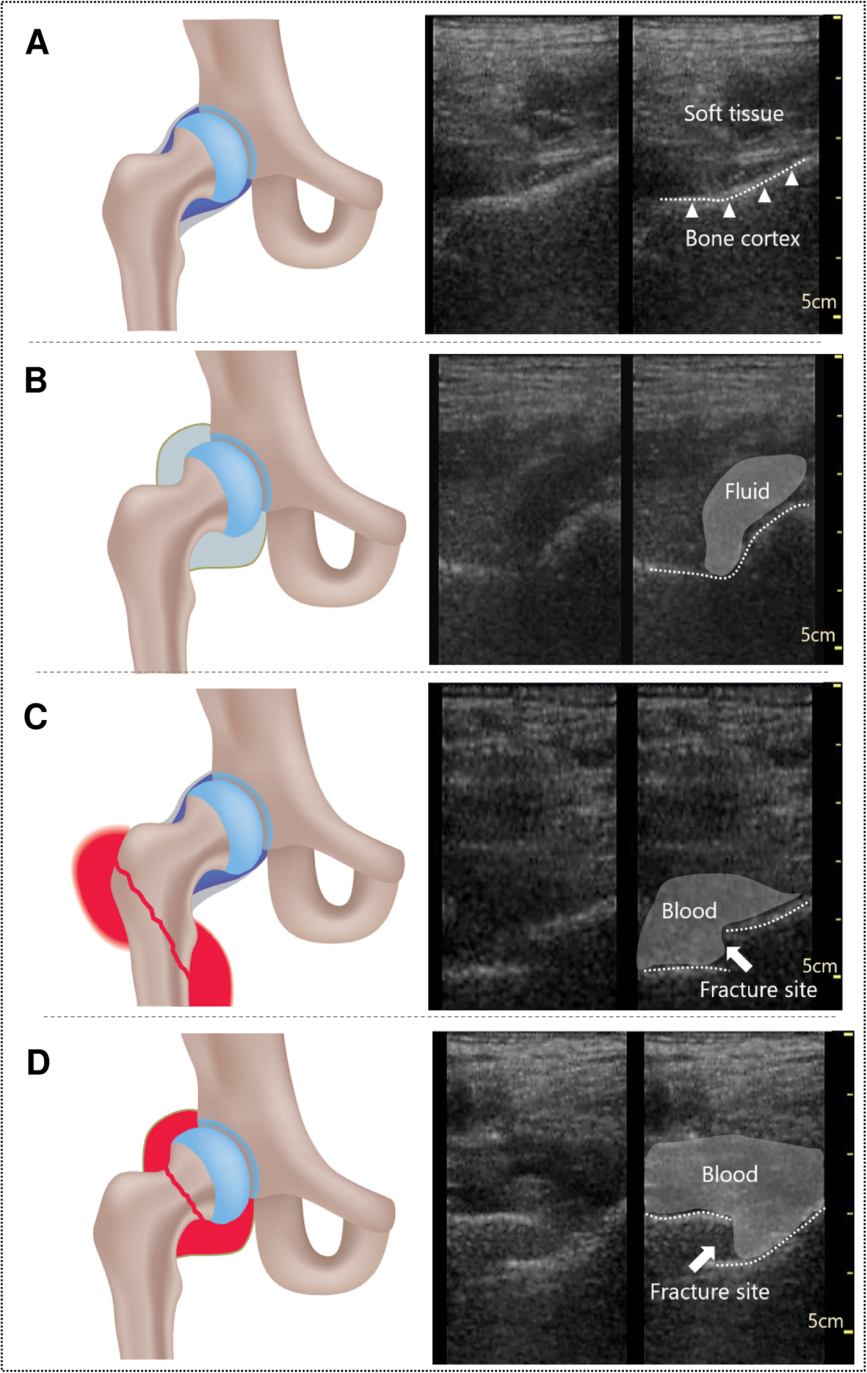
Schemas and ultrasound images of normal and abnormal hip joints. **a**, Normal. **b**, Fluid retention without fracture. **c**, Blood retention with trochanteric region fracture. **d**, Blood retention with neck fracture. This figure was drawn up by TK (author)

Of 52 patients, 46 were female (88.5%) with a mean age of 78.0 ± 12.2 years (mean ± standard deviation). Of 26 patients diagnosed with femoral proximal fractures, 14 had femoral neck fractures and 12 had trochanteric region fractures. No patient was diagnosed with femoral head fracture. Six patients had pubic and ischial fractures, and 6 had acute hip arthritis. The details and baseline characteristics are shown in Table 1. The typical findings of US are shown in Fig. 2.

**Table 1:** Patient characteristics and diagnoses (*N* = 52)

Age, mean (y) (SD)	78.0 (12.2)
Female, N (%)	46 (88.5)
Type of diagnosis, N (%)	
Fracture	32 (61.5)
Femoral proximal fracture	26 (50.0)
Femoral head fracture	0 (0.0)
Femoral neck fracture	14 (27.0)
Femoral trochanteric region fracture	12 (23.0)
Pubic and ischial fracture	6 (11.5)
Non-fracture	20 (38.5)
Acute hip arthritis	6 (11.5)
Aseptic arthritis	6 (11.5)
Septic arthritis	0 (0.0)
Unknown	14 (27.0)
Oral medication, n (%)	
Antiplatelet	11 (21.2)
Anticoagulants	3 (5.8)

*SD* standard deviation

(Visit with medical appointment 0%)

Cortical discontinuity was found in 25 of 26 (96%) patients with femoral proximal fractures. The Sn and Sp for identifying cortical discontinuity in femoral proximal fractures were 0.96 (95% confidence interval [CI], 0.80–1.00) and 0.92 (95% CI, 0.74–0.99), respectively. Joint fluid retention was found in 16 (62%) of 26 patients with femoral proximal fracture. The Sn and Sp for identifying joint fluid retention in femoral proximal fractures were 0.62 (95% CI, 0.41–0.80) and 0.77 (95% CI, 0.56–0.91), respectively. Joint fluid retention was found in 6 (100%) of 6 patients with acute hip arthritis. The Sn and Sp for identifying joint fluid retention in acute hip arthritis were 1.00 (95% CI, 0.42–1.00) and 0.65 (95% CI, 0.50–0.79), respectively. Either cortical discontinuity or joint fluid retention was found in 31 (97%) of 32 patients with femoral proximal fractures or acute hip arthritis. The Sn and Sp for identifying either cortical discontinuity or joint fluid retention in femoral proximal fractures or acute hip arthritis were 0.97 (95% CI, 0.84–1.00) and 0.90 (95% CI, 0.68–0.99), respectively (Table 2).

**Table 2:** Diagnostic accuracy of ultrasound findings for femoral proximal fracture and acute hip arthritis

US findings	Diagnosis	Sn (95% CI)	Sp (95% CI)	PPV (95% CI)	NPV (95% CI)
Cortical discontinuity	Femoral proximal fracture	0.96 (0.80–1.00)	0.92 (0.74–0.99)	0.93 (0.76–0.99)	0.96 (0.80–1.00)
Joint fluid retention	Femoral proximal fracture	0.62 (0.41–0.80)	0.77 (0.56–0.91)	0.73 (0.50–0.89)	0.67 (0.47–0.83)
	Acute hip arthritis	1.00 (0.42–1.00)	0.65 (0.50–0.79)	0.27 (0.11–0.50)	1.00 (0.83–1.00)
Cortical discontinuity or joint fluid retention	Femoral proximal fracture or acute hip arthritis	0.97 (0.84–1.00)	0.90 (0.68–0.99)	0.94 (0.80–0.99)	0.95(0.74–1.00)

*US* ultrasound, *Sn* sensitivity, *Sp* specificity, *PPV* positive predictive value, *NPV* negative predictive value, *CI* confidence interval

## Discussion

### Cortical discontinuity

A negative finding of cortical discontinuity on PsUS is useful in ruling out femoral proximal fractures (Sn, 0.96), whereas the presence of cortical discontinuity is suggestive of femoral proximal fractures (Sp, 0.92). The diagnostic accuracy of cortical discontinuity for femoral proximal fractures was comparable to that seen in a report using conventional US for fractures in various parts of the body (Sn, 0.94; Sp, 0.92) [3]. Furthermore, the diagnostic accuracy of cortical discontinuity for femoral proximal fractures was comparable to that of radiography (Sn, 0.97–0.98; Sp, 1.00) [16, 17]. The diagnostic accuracy of cortical discontinuity was equal to or more than that of the patellar-pubic percussion test (Sn, 0.79–0.96), which was reported as one of the most representative physical examinations to identify fractures [18, 19]. However, physicians should be attentive to false-negative findings when they cannot detect cortical discontinuity (e.g., localized fracture on the greater trochanter) and false-positive findings when they can only detect cortical discontinuity (e.g., misinterpretation of an acetabular head gap to cortical discontinuity) (Additional file 1 and Additional file 2).

### Joint fluid retention

A negative finding of joint fluid retention on PsUS is useful to rule out acute hip arthritis (Sn, 1.00); however, it cannot effectively rule out femoral proximal fracture. Positive findings of joint fluid retention on PsUS lead physicians to consider femoral neck fracture (positive predictive value, 0.73) rather than acute hip arthritis (positive predictive value, 0.27). The diagnostic accuracy of joint fluid retention for femoral neck fracture (Sp, 0.77; 95% CI, 0.56–0.91) is comparable to that of the fat pad sign that suggests joint swelling on radiographic finding (Sp, 0.86; cutoff point, 1.5 mm) [20]. In addition, physicians can increase the diagnostic accuracy by repeated US examinations with no radiation (i.e., to decrease false-negative finding of no joint fluid retention in the hip joint) [14, 21]. To diagnose acute septic or aseptic hip arthritis, joint fluid needle aspiration is recommended [1], which may develop complications, such as vascular or neurological injuries. In this study, we performed joint fluid needle aspiration in 2 of 6 patients (33%) who had acute hip arthritis. The final diagnosis for all the 6 patients was acute aseptic hip arthritis (100%).

### Combination of cortical discontinuity and joint fluid retention

PsUS finding of neither cortical discontinuity nor joint fluid retention is useful to rule out femoral proximal fractures and acute hip arthritis. The Sn of either cortical discontinuity or joint fluid retention was 0.97 for femoral proximal fracture or acute hip arthritis. Our study included 6 pubic and ischial fractures (12%); nevertheless, cortical discontinuity or joint fluid retention could not rule out other fractures, except femoral proximal fractures. A previous study to diagnose acute hip pain after post-traumatic injuries with normal radiographic finding showed 8 pubic and ischial fractures (27%), of which 4 (50%) were identified using conventional US [22]. Therefore, physicians should consider pubic and ischial fractures when they are unable to detect cortical discontinuity using US for patients with acute hip pain. In addition to pubic and ischial fractures, physicians should also consider other diseases, such as vascular abnormalities of the hip, soft-tissue abnormalities, and neurogenic causes [23], which were not seen in our study.

### Diagnostic accuracy of PsUS

We adopted a new unified approach of PsUS probe handling to evaluate femoral proximal fractures and acute hip arthritis (Fig. 2). Recent studies indicated that the accuracy among beginners was comparable to that among well-skilled US examiners for musculoskeletal examinations after a short training of 3 h for use of US [24] or stepwise from off-the-job to on-the-job educational training for 4 months [25]. Nevertheless, US demonstrates lower intra- and inter-rater reliability than radiography, CT, or MRI because US imaging depends on the examiner’s skill [26–28]. In this study, antalgic positions caused by femoral proximal fractures and acute hip arthritis were sometimes more difficult to examine using PsUS, especially in cases of excessive flexion and internal rotation of the hip joints. In this situation, the diagnostic sensitivity of PsUS might decrease. Furthermore, there could be heuristic bias in that the examiner might estimate the higher possibility of fracture if the patient complained of severe pain when the examiner performed PsUS. Therefore, to reach adequate intra-rater reliability, the PsUS examiner (TA) in our study was well-trained for the unified approach before the study started. Further evaluation of the intra- and inter-rater reliability of the approach should be performed in the future.

The diagnostic accuracy of US depends on the type of US. Higher-quality US imaging may have a higher diagnostic accuracy. Recently, portable and high-image-quality PsUS devices have been rapidly developing around the world. Therefore, we believe that the diagnostic accuracy of PsUS for femoral proximal fractures and acute hip arthritis will further improve.

### Clinical perspective

PsUS decreases unnecessary referral to orthopedic specialists by optimizing medical examination for acute hip pain. Physicians are recommended to take radiographs when fracture or dislocation is suspected [1]. However, if a physician cannot use these standard diagnostic devices in rural clinics or nursing homes, PsUS findings of the absence of cortical discontinuity or joint fluid retention can help rule out femoral proximal fractures or acute hip arthritis (Sn, 0.97), leading to a decrease in unnecessary outpatient visits, referrals, and imaging. Physicians must consider referral to orthopedic specialists when they confirm cortical discontinuity and joint fluid retention due to hip joint fractures on US.

### Limitations

Our study has the following limitations. First, we could not validate the reliability of PsUS for the patient’s body weight because we could not use a stretcher scale with beds. The quality of the US image depends on the patient’s body shape, especially the thickness of the subcutaneous tissue. Second, we conducted this study at a single medical facility in Japan. Therefore, further studies with larger samples are warranted to generalize our results. Third, we used a Vscan dual probe as the PsUS. We did not evaluate other PsUS devices. Therefore, the diagnostic accuracy of other PsUS devices is unknown. Fourth, the US image with the linear probe may have the same accuracy as that of a convex or sector probe.

## Conclusions

The PsUS finding of the absence of cortical discontinuity or joint fluid retention in the hip is useful in ruling out femoral proximal fractures and acute hip arthritis. PsUS could contribute to the initial assessment of acute hip pain in the elderly, especially in the primary care setting.

## Supplementary information

**Additional file 1.** Femoral head (left side) and acetabulum (right side) as a normal anatomical structure.

**Additional file 2.** Supplemental movie, rotation of femoral head (normal).

## Data Availability

The anonymized datasets used and/or analyzed during the current study are available from the corresponding author on reasonable request.

## Abbreviations

CT: computed tomography
MRI: magnetic resonance imaging
PsUS: pocket-sized ultrasound
Sn: sensitivity
Sp: specificity
US: ultrasound devices
