# Supplementary figures and images for: Initial assessment of femoral proximal fracture and acute hip arthritis using pocket-sized ultrasound: a prospective observational study in a primary care setting in Japan
Source: BMC Musculoskelet Disord. 2020 May 11;21:291. doi: 10.1186/s12891-020-03326-x (PMC7216341; doi:10.1186/s12891-020-03326-x)

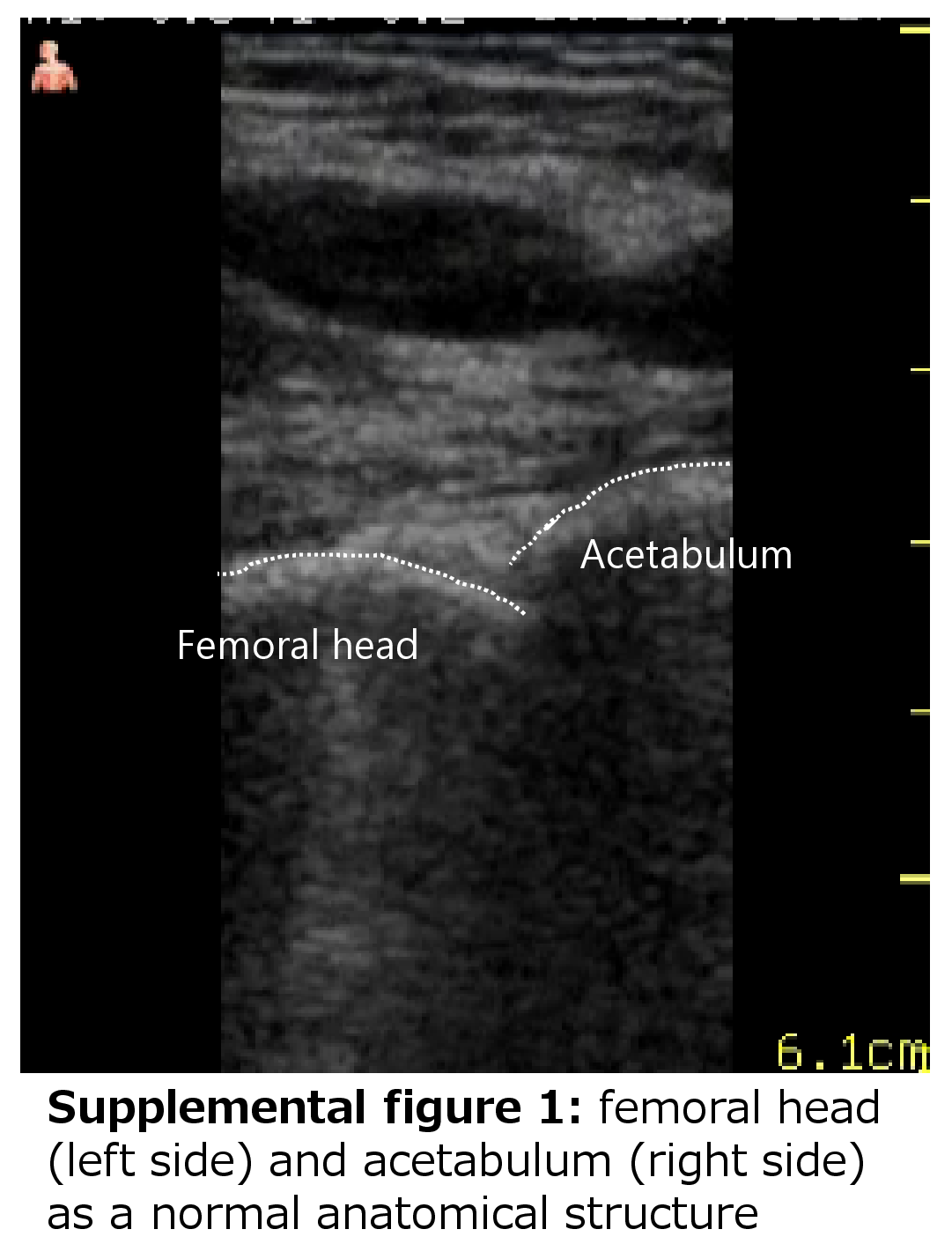

Supplement: Supplementary file 1 — Additional file 1. Femoral head (left side) and acetabulum (right side) as a normal anatomical structure. [file 12891_2020_3326_MOESM1_ESM.tiff]
